# Supplementary material for: High-risk HPV genotypes in Zimbabwean women with cervical cancer: Comparative analyses between HIV-negative and HIV-positive women
Source: PLoS One. 2021 Sep 28;16(9):e0257324. doi: 10.1371/journal.pone.0257324 (PMC8478215; doi:10.1371/journal.pone.0257324)
Supplement: S1 Table — (PDF) [file pone.0257324.s001.pdf]

	Odds ratio	95% CI	P value
**HPV16**			
**Age**	1.0	0.9 – 1.0	0.686
**Sexual debut**	0.8	0.6 - 1.2	0.304
**Parity**	1.7	0.8 – 3.6	0.146
**STI history**	0.7	0.1 – 7.7	0.751
**HIV**	1.0	0.6 – 1.5	0.839
**HPV18**			
**Age**	12.5	-13 – 38	0.333
**Sexual debut**	-6.7	-22 – 9	0.398
**Parity**	1.3	0.5 – 3.5	0.627
**STI history**	0.2	-0.0 – 0.09	0.489
**HIV**	1.2	0.8 – 1.9	0.446
**HPV31**			
**Age**	1.0	1.0 – 1.1	0.808
**Sexual debut**	0.9	0.7 – 1.1	0.340
**Parity**	0.8	0.6 – 1.0	0.063
**STI history**	2.3	0.8 – 6.3	0.117
**HIV**	0.8	0.3 – 2.3	0.664
**HPV33**			
**Age**	1.0	0.9 – 1.0	0.391
**Sexual debut**	1.0	0.8 – 1.2	0.746
**Parity**	1.0	0.8 – 1.3	0.668
**STI history**	1.2	0.5 – 3.0	0.684
**HIV**	0.4	0.2 – 1.1	0.079
**HPV35**			
**Age**	1.2	0.9 – 1.5	0.341
**Sexual debut**	0.7	0.3 – 1.4	0.312
**Parity**	1.0	-4.0 – 6.0	0.683
**STI history**	0.0	-0.0 – 0.10	0.576
**HIV**	0.8	0.5 – 1.3	0.447
**HPV39**			
**Age**	1.0	1.0 – 1.1	0.307
**Sexual debut**	1.2	0.9 – 1.4	0.148
**Parity**	1.0	0.7 – 1.3	0.761
**STI history**	1.4	0.4 – 5.1	0.649
**HIV**	2.1	0.5 – 8.8	0.295
**HPV45**			
**Age**	1.1	0.82 – 1.44	0.551
**Sexual debut**	0.9	0.60 – 1.24	0.414
**Parity**	1.2	0.45 – 3.39	0.681

**STI history**	0.3	-0.76 – 1.33	0.566
**HIV**	1.1	0.4 – 2.8	0.854
**HPV51**			
**Age**	1.1	0.88 -1.31	0.480
**Sexual debut**	0.7	0.25 – 1.7	0.374
**Parity**	1.1	0.36 – 3.54	0.834
**STI history**	1.0	-0.24 – 0.52	0.424
**HIV**	1.9	0.7 - 4.7	0.179
**HPV52**			
**Age**	1.1	0.85 – 1.52	0.382
**Sexual debut**	0.9	0.50 – 1.50	0.579
**Parity**	0.5	0.09 – 3.18	0.487
**STI history**	0.3	-0.21 – 0.71	0.242
**HIV**	0.8	0.2 – 2.9	0.731
**HPV58**			
**Age**	1.0	1.0 – 1.1	0.209
**Sexual debut**	1.0	0.9 – 1.2	0.800
**Parity**	1.0	0.8 – 1.2	0.661
**STI history**	0.8	0.3 – 2.0	0.573
**HIV**	2.5	1.0 – 6.2	0.051
**HPV58**			
**Age**	1.1	0.86 – 1.44	0.419
**Sexual debut**	0.7	0.25 – 1.67	0.374
**Parity**	1.1	0.44 – 2.79	0.822
**STI history**	0.0	-0.14 – 0.23	0.611
**HIV**	1.6	0.8 – 3.4	0.194
**HPV59**			
**Age**	0.1	-0.08 – 0.24	0.198
**Sexual debut**	1.0	0.88 – 1.22	0.650
**Parity**	-0.8	-2.53 – 1.03	0.272
**STI history**	0.7	-0.70 – 2.03	0.219
**HIV**	3.7	0.4 – 35.72	0.262
**HPV66[a]**			
**Age**	1.1	0.81 – 1.57	0.473
**Sexual debut**	1.0	0.81 – 1.21	0.959
**Parity**	1.0	-0.31 – 0.54	0.369
**HIV**	1.2	0.1 – 19.47	0.894
**HPV68**			
**Age**	1.1	0.83 – 1.39	0.594
**Sexual debut**	1.1	0.91 – 1.29	0.382
**Parity**	1.2	0.49 – 2.94	0.685
**STI history**	0.3	-0.61 – 1.28	0.420

**HIV**	1.5	0.40 – 5.79	0.535
**Number of HPV infections**			
**Age**	1.0	0.95 – 1.04	0.802
**Sexual debut**	0.9	0.82 – 1.01	0.085
**Parity**	1.0	0.76 – 1.20	0.690
**STI history**	0.5	0.14 – 1.62	0.239
**HIV**	1.5	0.42 – 5.13	0.545

Note: STI, sexually transmitted infection; CI, confidence interval; a – no individual with HPV genotype had STI history
