# Supplementary material for: High-risk HPV genotypes in Zimbabwean women with cervical cancer: Comparative analyses between HIV-negative and HIV-positive women
Source: PLoS One. 2021 Sep 28;16(9):e0257324. doi: 10.1371/journal.pone.0257324 (PMC8478215; doi:10.1371/journal.pone.0257324)
Supplement: S2 Table — (PDF) [file pone.0257324.s002.pdf]

S2 Table. The number of individuals harbouring specific HPV genotypes stratified by HIV status.

		Number of HPVs (n)					
**HPV genotype**	**HIV status**	**1 ***	**2**	**3**	**4**	**5**	**6**
16	-	19	18	5	1	0	0
	+	15	13	3	1	1	0
18	-	13	12	1	0	1	1
	+	6	13	1	0	0	0
31	-	1	0	1	2	0	0
	+	1	0	0	0	0	1
33	-	2	0	1	2	0	0
	+	2	0	0	0	0	0
35	-	15	15	3	0	1	0
	+	3	10	3	0	1	1
39	-	0	2	0	1	0	0
	+	0	1	0	0	0	0
45	-	1	0	2	3	0	0
	+	0	0	1	1	0	0
51	-	0	0	1	0	1	0
	+	1	1	1	1	1	1
52	-	1	2	1	0	0	0
	+	0	3	1	0	0	1
56	-	0	0	0	1	0	0
	+	0	0	1	0	1	0
58	-	0	2	1	1	1	0
	+	0	2	0	0	1	0
59	-	0	0	0	2	0	0
	+	2	0	1	1	0	0
66	-	0	0	0	1	0	0
	+	0	0	1	0	0	0
68	-	2	0	1	0	1	0
	+	1	0	1	1	2	1
**TOTAL**		85	94	31	19	12	6
