# Supplementary material for: High-risk HPV genotypes in Zimbabwean women with cervical cancer: Comparative analyses between HIV-negative and HIV-positive women
Source: PLoS One. 2021 Sep 28;16(9):e0257324. doi: 10.1371/journal.pone.0257324 (PMC8478215; doi:10.1371/journal.pone.0257324)
Supplement: S3 Table — (PDF) [file pone.0257324.s003.pdf]

HPV genotypes*	OR (95% CI)	p
16/18	0.3 (0.1-0.5)	**<0.01**
16/33	0.4 (0.2-1.0)	0.05
16/35	3.6 (1.8-7.2)	**<0.01**
35/51	8.8 (2.3-34.3)	**<0.01**
35/52	0.5 (0.1-4.3)	0.50
39/51	4.2 (0.7-25.9)	0.12
39/56	9.5 (1.1-82.9)	**0.04**

*multivariate analysis for the co-segregation of HPV genotypes, taking into consideration age, history of STI, parity, HIV status, age of sexual debut, tumour histology.
