# Supplementary material for: High-risk HPV genotypes in Zimbabwean women with cervical cancer: Comparative analyses between HIV-negative and HIV-positive women
Source: PLoS One. 2021 Sep 28;16(9):e0257324. doi: 10.1371/journal.pone.0257324 (PMC8478215; doi:10.1371/journal.pone.0257324)
Supplement: Attachment — Submitted filename: PLOS Repsonses to reviewers_14Apr2021.docx [file pone.0257324.s004.docx]

***Response to reviewer comments: PONE-D-21-04071***

*28 April 2021*

Dear Professor, Joakim Dilner,

Thank you for reviewing our manuscript PONE-D-21-04071- entitled “**High-Risk HPV genotypes in Zimbabwean women with cervical cancer: Comparative Analyses between HIV-negative and HIV-positive women”** and providing us with constructive criticism on how to improve the statistical analysis and the scientific soundness of the work. Below are our responses to each of the reviewers’ and editor’s comments.

**Reviewer #1:**

1. **Throughout the text “despite” is often used when it should be “irrespective of” or “regardless of” for example third line in result-section of abstract**

Thank you for pointing this out. The authors have edited the document accordingly, and replaced the word “despite” with other terminologies

2. **Methods: I would suggest using regression analyses for Table 2, 3 and 4 rather than Chi2. Either Poisson regression for prevalence ratios or Logistic regression for odds ratios.**

Thank you for this suggestion. The re-analysis for Tables 2 and 3 was performed, as per the reviewer’s recommendations. Data generated for Table 2 was tested using Poisson’s regression, while Table 3 was analysed using logistic regression. However, Table 4 compares absolute frequencies for the HPV genotypes as recorded by an established database, the HPV Information Centre (https://hpvcentre.net) and so the best analyses to compare these frequencies is using the Chi2 statistical methods.

**Table 2: HR-HPV genotypes by HIV status (n=258) and the HPV phylogenetic classification.**

|  | **Frequency (Proportion)** | | |  |  |
| --- | --- | --- | --- | --- | --- |
| **HPV genotype** | **All** | **HIV negative** | **HIV positive** | **IRR (95% CI)** | **p value** |
| Negative | 10 (0.04) | 6 (0.02) | 4 (0.02) | 0.40 (0.22 – 1.00) | 0.443 |
| 16^a^ | 123 (0.48) | 66 (0.26) | 57 (0.22) | 0.97 (0.69 - 1.37) | 0.881 |
| 18^b^ | 65 (0.25) | 34 (0.13) | 31 (0.12) | 1.11 (0.78 – 1.57) | 0.573 |
| 31^a^ | 17 (0.07) | 8 (0.03) | 9 (0.04) | 1.06 (0.57 – 1.96) | 0.854 |
| 33^a^ | 25 (0.10) | 18 (0.07) | 7 (0.03) | 0.81 (0.49 – 1.34) | 0.416 |
| 35^a^ | 68 (0.26) | 40 (0.16) | 28 (0.10) | 0.90 (0.63 – 1.29) | 0.574 |
| 39^b^ | 10 (0.04) | 3 (0.01) | 7 (0.03) | 1.42 (0.67 – 3.05) | 0.361 |
| 45^b^ | 14 (0.05) | 8 (0.03) | 6 (0.02) | 1.05 (0.53 – 2.06) | 0.892 |
| 51^c^ | 11 (0.04) | 5 (0.02) | 6 (0.02) | 1.35 (0.75 – 2.45) | 0.316 |
| 52^a^ | 9 (0.04) | 5 (0.02) | 4 (0.02) | 0.88 (0.33 – 2.38) | 0.799 |
| 56^d^ | 6 (0.02) | 2 (0.0) | 4 (0.02) | 1.11 (0.41 – 2.99) | 0.843 |
| 58^a^ | 23 (0.11) | 9 (0.05) | 14 (0.05) | 1.28 (0.78 – 2.09) | 0.334 |
| 59^b^ | 3 (0.01) | 0 (0.00) | 3 (0.01) | 1.67 (0.53 – 5.24) | 0.380 |
| 66^d^ | 1 (0.0) | 0 (0.00) | 1 (0.01) | 1.1 (0.15 – 7.89) | 0.922 |
| 68^b^ | 7 (0.03) | 2 (0.01) | 5 (0.02) | 1.23 (0.51 – 3.01) | 0.645 |

**IRR= incidence rate ratio computed from the Poisson regression analysis**

**a= Human papillomavirus alpha 9 species; b= Human papillomavirus alpha 7 species; c= Human papillomavirus alpha 5 species; d= Human papillomavirus alpha 6 species.**

**Table 3: Multiple HR-HPV infections, stratified by HIV status (n=163).**

| **Number of HPVs found together** | **Frequency (Proportion)** | | |  |  |
| --- | --- | --- | --- | --- | --- |
|  | **All** | **HIV negative** | **HIV positive** | **OR (95% CI)** | **p-value** |
| 2 | 94 (0.59) | 45 (0.28) | 50 (0.31) | 1.65 (0.46 – 5.98) | 0.443 |
| 3 | 31 (0.19) | 16 (0.10) | 15 (0.09) | 2.10 (0.55 –8.01) | 0.277 |
| 4 | 19 (0.12) | 11 (0.07) | 8 (0.05) | 0.80 (0.16 – 4.02) | 0.782 |
| 5 | 12 (0.08) | 6 (0.04) | 6 (0.04) | 1.75 (0.08- 36.28) | 0.718 |
| 6 | 6 (0.04) | 4 (0.03) | 2 (0.01) | 1.2 (0.34 – 4.41) | 0.756 |

3. **Results: I suggest categorizing by HIV status in Figure 2.**

The authors appreciate this recommendation and have presented the data as recommended, please see below.

**Figure 2: The HPV mono infection distribution in women with confirmed cervical cancer (n=85).**

4. **Results: For better understanding of Figure 3A and Figure 3B it might be better if the absolute numbers of certain genotype combinations were presented. I lack an explanation as to how to assess the odds ratios (eg OR increased at 3.3 for the most common combination 16/35 but decreased at OR 0.3 for the second most common combination 16/18. Is 16 reference?).**

Thank you for indicating this, a supplementary table was added to this effect (Supplementary Table 2). Logistic regression analysis was performed to estimate the co-occurrence by adding the two specific HPV genotypes as the predictor and co-variate. For example, where HPV16/18 are analysed, it indicates that HPV16 is the predictor, and HPV18 is the covariate. In the comparative analysis, the reference is the whole dataset, less the HPV genotypes being analysed for co-occurrence.

| **Supplementary Table 2: HPV co-occurrence regression analysis, without HIV status, and with HIV status as a co-variate.** | | | | | |
| --- | --- | --- | --- | --- | --- |
| **Composite Genotypes** | **N (proportion)** | **OR (95% CI)^a^** | **p^a^** | **OR (95% CI)^b^** | **p^b^** |
| 16/18 | 44 (0.17) | 0.3 (0.2- 0.5) | **<0.001** | 0.3 (0.2- 0.5) | **<0.001** |
| 16/31 | 14 (0.05) | 0.9 (0.4-2.2) | 0.824 | 1 (0.4- 2.1) | 0.773 |
| 16/33 | 18 (0.07) | 0.3 (0.2 – 0.6) | **0.001** | 0.3 (0.2-0.7) | **0.001** |
| 16/35 | 83 (0.32) | 3.3 (1.9 – 5.7) | **<0.001** | 3.7 (2.1-6.4) | **<0.001** |
| 16/39 | 6 (0.02) | 0.7 (0.2 – 2.3) | 0.555 | 0.6 (0.2 – 2.9) | 0.358 |
| 16/45 | 14 (0.05) | 1.7 (0.6 – 4.8) | 0.325 | 1.7 (0.6 – 5.0) | 0.331 |
| 16/51 | 15 (0.06) | 1.8 (0.6 – 5.2) | 0.257 | 1.6 (0.6 – 4.6) | 0.386 |
| 16/52 | 5 (0.02) | 0.6 (0.2 – 2.0) | 0.391 | 0.6 (0.2 – 2.2) | 0.426 |
| 16/56 | 7 (0.03) | 4.2 (0.5 – 34.8) | 0.181 | 4.3 (0.5 – 36.1) | 0.182 |
| 16/58 | 23 (0.09) | 1.4 (0.6 – 3.2) | 0.410 | 1.4 (0.6 – 3.2) | 0.410 |
| 16/59 | 1 (0.00) | 0.2 (0.0 – 1.9) | 0.154 | 0.1 (0.0 – 1.3) | 0.083 |
| 18/31 | 5 (0.02) | 0.6 (0.21 – 1.6) | 0.285 | 0.5 (0.2 – 1.5) | 0.250 |
| 18/33 | 12 (0.05) | 0.7 (0.4 – 1.5) | 0.422 | 0.8 (0.4 – 1.7) | 0.567 |
| 18/35 | 26 (0.10) | 0.6 (0.4 – 1.0) | 0.068 | 0.6 (0.4 – 1.1) | 0.089 |
| 18/39 | 1 (0.00) | 0.2 (0.0 – 1.6) | 0.135 | 0.2 (0.0- 1.3) | 0.088 |
| 18/45 | 3 (0.01) | 0.4 (0.1 – 1.3) | 0.134 | 0.4 (0.1 – 1.3) | 0.116 |
| 18/51 | 7 (0.03) | 1.2 (0.4 – 3.0) | 0.753 | 1.0 (0.4 – 2.7) | 0.998 |
| 18/52 | 3 (0.01) | 0.9 (0.2 – 3.6) | 0.899 | 1.0 (0.2 – 4.0) | 0.961 |
| 18/56 | 4 (0.02) | 2.2 (0.5 – 8.9) | 0.275 | 2.2 (0.5 – 9.3) | 0.291 |
| 18/58 | 11 (0.04) | 1.1 (0.5 – 2.5) | 0.745 | 1.0 (0.5 – 2.2) | 0.979 |
| 18/59 | 2 (0.01) | 2.2 (0.3 – 15.6) | 0.443 | 1.7 (0.2 – 12.5) | 0.621 |
| 18/68 | 1 (0.00) | 0.3 (0.0 – 2.1) | 0.207 | 0.2 (0.0 – 1.8) | 0.164 |
| 31/33 | 1 (0.00) | 0.3 (0.0 – 2.1) | 0.210 | 0.3 (0.0 – 1.9) | 0.183 |
| 31/35 | 7 (0.03) | 0.9 (0.3 – 2.2) | 0.751 | 0.9 (0.4 – 2.3) | 0.823 |
| 31/45 | 3 (0.01) | 0.3 (0.0 - 1.9 ) | 0.183 | 2.5 (0.7 -9.7) | 0.169 |
| 31/51 | 2 (0.01) | 1.4 (0.3 – 6.6) | 0.647 | 1.2 (0.3 – 5.8) | 0.784 |
| 31/52 | 3 (0.01) | 6.0 (1.4 – 25.1) | **0.014** | 6.8 (1.6 – 29.2) | **0.010** |
| 31/56 | 1 (0.00) | 1.8 (0.2 – 15.5) | 0.582 | 1.8 (0.2 – 15.4) | 0.608 |
| 31/58 | 1 (0.00) | 0.4 (0.0 – 2.9) | 0.348 | 0.3 (0.0 – 2.6) | 0.291 |
| 31/68 | 1 (0.00) | 1.6 (0.2 – 13.3) | 0.668 | 1.5 (0.2 – 12.4) | 0.734 |
| 33/35 | 8 (0.03) | 0.4 (1.2 - 0.9) | **0.019** | 0.4 (0.2 -0.9) | **0.024** |
| 33/39 | 1(0.00) | 0.6 (0.1 – 4.7) | 0.610 | 0.5 (0.1 – 3.9) | 0.494 |
| 33/45 | 4 (0.02) | 1.6 (0.5 – 5.2) | 0.404 | 1.6 (0.5 – 5.3) | 0.418 |
| 33/51 | 2 (0.01) | 0.6 (0.1 – 2.9) | 0.560 | 0.5 (0.1 – 2.5) | 0.432 |
| 33/52 | 1 (0.00) | 0.6 (0.1 – 5.3) | 0.685 | 0.7 (0.0 – 5.6) | 0.720 |
| 33/58 | 5 (0.02) | 1.1 (0.4 – 3.0) | 0.845 | 1.0 (0.4 – 2.7) | 0.977 |
| 33/68 | 3 (0.01) | 2.9 (0.7 – 12.6) | 0.150 | 3.1 (0.7 – 12.9) | 0.120 |
| 35/39 | 2 (0.01) | 0.4 (0.1 – 2.0) | 0.288 | 0.3 (0.1 -1.7) | 0.185 |
| 35/45 | 5 (0.02) | 0.4 (0.1 – 2.0) | 0.288 | 0.7 (0.2 -2.0) | 0.462 |
| 35/51 | 13 (0.05) | 4.1 (1.6 – 10.6) | **0.004** | 4.0 (1.4 – 10.0) | **0.008** |
| 35/52 | 2 (0.01) | 0.5 (0.1 – 2.3) | 0.369 | 0.5 (0.1 – 2.5) | 0.394 |
| 35/56 | 3 (0.01) | 1.2 (0.3 – 5.1) | 0.805 | 1.1 (0.3 – 5.1) | 0.851 |
| 35/58 | 12 (0.05) | 1.2 (0.6 – 2.6) | 0.608 | 1.1 (0.5 – 2.4) | 0.832 |
| 35/66 | 1 (0.00) | 2 (0.1 – 32.3) | 0.625 | 2.0 (0.1 – 35.0) | 0.639 |
| 39/45 | 1 (0.00) | 1.6 (0.2 – 12.9) | 0.677 | 1.5 (0.2 -12.9) | 0.690 |
| 39/51 | 3 (0.01) | 6.2 (1.5-25.7) | **0.011** | 5.5 (1.3 – 23.1) | **0.019** |
| 39/56 | 2 (0.01) | 11 1.9 -62) | **0.007** | 10.9 (2.0 - 64.3) | **0.008** |
| 39/58 | 1 (0.00) | 0.9 (0.1 -7.0) | 0.894 | 1 (0.1 – 6.3) | 0.810 |
| 39/59 | 1 (0.00) | 9.9 (0.9 - 103) | 0.056 | 7.8 (0.7 – 84.6) | 0.090 |
| 39/68 | 1 (0.00) | 3.4 (0.4 – 30.1) | 0.279 | 3.6 (0.5 – 30.0) | 0.280 |
| 45/52 | 1 (0.00) | 1.9 (0.2 – 15.9) | 0.569 | 1.9 (0.2 – 15.9) | 0.569 |
| 45/56 | 1 (0.00) | 2.2 (0.2 – 19.3) | 0.479 | 2.2 (0.2 – 19.3) | 0.479 |
| 45/58 | 1 (0.00) | 0.4 (0.0 – 3.2) | 0.400 | 0.4 (0.0 – 3.2) | 0.400 |
| 45/59 | 1 (0.00) | 4.2 (0.4 – 43.8) | 0.229 | 4.2 (0.4 – 43.8) | 0.229 |
| 45/68 | 1 (0.00) | 2.0 (0.2 - 16.6) | 0.533 | 1.8 (0.2 – 15.6) | 0.591 |
| 51/52 | 1 (0.00) | 1.6 (0.2 – 13.7) | 0.643 | 1.4 (0.2 – 12.0) | 0.740 |
| 51/56 | 2 (0.01) | 5.3 (1.0 – 28.0) | 0.051 | 5.3 (1.0 – 28.9) | 0.056 |
| 51/58 | 2 (0.01) | 1.0 (0.2 – 4.4) | 0.965 | 0.9 (0.2 – 3.9) | 0.847 |
| 58/68 | 2 (0.01) | 2.3 (1.0 – 11.2) | 0.300 | 2.4 (0.5- 12.5) | 0.296 |
| 59/68 | 2 (0.01) | 42 (5.3 – 349.4) | **0.001** | 35.4 (4.2 - 301) | **0.001** |
| 66/68 | 1 (0.00) | 37 (2.2 – 565.6) | **0.013** | 40 (2-766.4) | **0.014** |
| **a- analyses with no consideration of HIV status; b- analyses with HIV status as a co-variate** | | | | | |

5. **Results: It says 58% having 2 HR-HPVs in the text but 59% in Table 3.**

Thank you for this observation, this statement has been clarified based on re-analysis of the data, and the actual value is 59%.

6. **Supplementary Table 1. Suggest adding HIV as risk factor**

The authors appreciate this recommendation and have presented the data as recommended.

**Supplementary Table 1: Risk factors of HPV in association with the various HPV genotypes**

|  | **Odds ratio** | **95% CI** | **P value** |
| --- | --- | --- | --- |
| **HPV16** |  |  |  |
| Age | 1.0 | 0.9 – 1.0 | 0.686 |
| Sexual debut | 0.8 | 0.6 - 1.2 | 0.304 |
| Parity | 1.7 | 0.8 – 3.6 | 0.146 |
| STI history | 0.7 | 0.1 – 7.7 | 0.751 |
| HIV | 1.0 | 0.6 – 1.5 | 0.839 |
| **HPV18** |  |  |  |
| Age | 12.5 | -13 – 38 | 0.333 |
| Sexual debut | -6.7 | -22 – 9 | 0.398 |
| Parity | 1.3 | 0.5 – 3.5 | 0.627 |
| STI history | 0.2 | -0.0 – 0.09 | 0.489 |
| HIV | 1.2 | 0.8 – 1.9 | 0.446 |
| **HPV31** |  |  |  |
| Age | 1.0 | 1.0 – 1.1 | 0.808 |
| Sexual debut | 0.9 | 0.7 – 1.1 | 0.340 |
| Parity | 0.8 | 0.6 – 1.0 | 0.063 |
| STI history | 2.3 | 0.8 – 6.3 | 0.117 |
| HIV | 0.8 | 0.3 – 2.3 | 0.664 |
| **HPV33** |  |  |  |
| Age | 1.0 | 0.9 – 1.0 | 0.391 |
| Sexual debut | 1.0 | 0.8 – 1.2 | 0.746 |
| Parity | 1.0 | 0.8 – 1.3 | 0.668 |
| STI history | 1.2 | 0.5 – 3.0 | 0.684 |
| HIV | 0.4 | 0.2 – 1.1 | 0.079 |
| **HPV35** |  |  |  |
| Age | 1.2 | 0.9 – 1.5 | 0.341 |
| Sexual debut | 0.7 | 0.3 – 1.4 | 0.312 |
| Parity | 1.0 | -4.0 – 6.0 | 0.683 |
| STI history | 0.0 | -0.0 – 0.10 | 0.576 |
| HIV | 0.8 | 0.5 – 1.3 | 0.447 |
| **HPV39** |  |  |  |
| Age | 1.0 | 1.0 – 1.1 | 0.307 |
| Sexual debut | 1.2 | 0.9 – 1.4 | 0.148 |
| Parity | 1.0 | 0.7 – 1.3 | 0.761 |
| STI history | 1.4 | 0.4 – 5.1 | 0.649 |
| HIV | 2.1 | 0.5 – 8.8 | 0.295 |
| **HPV45** |  |  |  |
| Age | 1.1 | 0.82 – 1.44 | 0.551 |
| Sexual debut | 0.9 | 0.60 – 1.24 | 0.414 |
| Parity | 1.2 | 0.45 – 3.39 | 0.681 |
| STI history | 0.3 | -0.76 – 1.33 | 0.566 |
| HIV | 1.1 | 0.4 – 2.8 | 0.854 |
| **HPV51** |  |  |  |
| Age | 1.1 | 0.88 -1.31 | 0.480 |
| Sexual debut | 0.7 | 0.25 – 1.7 | 0.374 |
| Parity | 1.1 | 0.36 – 3.54 | 0.834 |
| STI history | 1.0 | -0.24 – 0.52 | 0.424 |
| HIV | 1.9 | 0.7 - 4.7 | 0.179 |
| **HPV52** |  |  |  |
| Age | 1.1 | 0.85 – 1.52 | 0.382 |
| Sexual debut | 0.9 | 0.50 – 1.50 | 0.579 |
| Parity | 0.5 | 0.09 – 3.18 | 0.487 |
| STI history | 0.3 | -0.21 – 0.71 | 0.242 |
| HIV | 0.8 | 0.2 – 2.9 | 0.731 |
| **HPV58** |  |  |  |
| Age | 1.0 | 1.0 – 1.1 | 0.209 |
| Sexual debut | 1.0 | 0.9 – 1.2 | 0.800 |
| Parity | 1.0 | 0.8 – 1.2 | 0.661 |
| STI history | 0.8 | 0.3 – 2.0 | 0.573 |
| HIV | 2.5 | 1.0 – 6.2 | 0.051 |
| **HPV58** |  |  |  |
| Age | 1.1 | 0.86 – 1.44 | 0.419 |
| Sexual debut | 0.7 | 0.25 – 1.67 | 0.374 |
| Parity | 1.1 | 0.44 – 2.79 | 0.822 |
| STI history | 0.0 | -0.14 – 0.23 | 0.611 |
| HIV | 1.6 | 0.8 – 3.4 | 0.194 |
| **HPV59** |  |  |  |
| Age | 0.1 | -0.08 – 0.24 | 0.198 |
| Sexual debut | 1.0 | 0.88 – 1.22 | 0.650 |
| Parity | -0.8 | -2.53 – 1.03 | 0.272 |
| STI history | 0.7 | -0.70 – 2.03 | 0.219 |
| HIV | 3.7 | 0.4 – 35.72 | 0.262 |
| **HPV66^a^** |  |  |  |
| Age | 1.1 | 0.81 – 1.57 | 0.473 |
| Sexual debut | 1.0 | 0.81 – 1.21 | 0.959 |
| Parity | 1.0 | -0.31 – 0.54 | 0.369 |
| HIV | 1.2 | 0.1 – 19.47 | 0.894 |
| **HPV68** |  |  |  |
| Age | 1.1 | 0.83 – 1.39 | 0.594 |
| Sexual debut | 1.1 | 0.91 – 1.29 | 0.382 |
| Parity | 1.2 | 0.49 – 2.94 | 0.685 |
| STI history | 0.3 | -0.61 – 1.28 | 0.420 |
| HIV | 1.5 | 0.40 – 5.79 | 0.535 |
| **Number of HPV infections** |  |  |  |
| Age | 1.0 | 0.95 – 1.04 | 0.802 |
| Sexual debut | 0.9 | 0.82 – 1.01 | 0.085 |
| Parity | 1.0 | 0.76 – 1.20 | 0.690 |
| STI history | 0.5 | 0.14 – 1.62 | 0.239 |
| HIV | 1.5 | 0.42 – 5.13 | 0.545 |

**Note:** STI, sexually transmitted infection; CI, confidence interval; a – no individual with HPV genotype had STI history

**Reviewer #2:**

1. **It would be informative to provide a brief description of the HPV vaccination program and uptake in Zimbabwe early in the introduction.**

The coverage for the HPV prophylactic vaccines is in its infancy in most developing countries, where the coverage is estimated to be more than 12-fold lower than developed countries [10]. As a matter of interest countries such as Zambia, Malawi and Zimbabwe, where the highest global cervical cancer cases are recorded, are still in the pilot and early implementation phases of the national HPV vaccination programs [11-12].

2. **Could the authors clarify the reasons of only including cervical cancer diagnosed at stage 1b-4.**

Thank you for noting this, we have clarified the selection criteria for our cohort. Our study recruited individuals from an outpatient radiotherapy and chemotherapy treatment facility, therefore, only patients seeking this type of care attend the clinics. Consequently, this excludes patients receiving surgical interventions only, or are receiving palliative care, because these individuals receive care from other oncological units in the hospital. The methods describe this as:

The participants from the current study were nested in a pharmacogenomics of cervical cancer study, aiming to recruit women who were newly diagnosed with cervical cancer, and were eligible to receive radical therapy. Potential study participants were identified through the RTC hospital registry. To be considered eligible for this study, women had to be ≥18 years, with histologically confirmed diagnoses of invasive cervical cancer staged between 1b – 4A, and were earmarked for curative anti-cancer therapy. Individuals who were diagnosed with resectable (stage 1A) disease that did not require chemo/radio- therapies, or with advanced disease that required palliative care (stage 4B) were excluded from the study, because these individuals were referred for care outside of the RTC.

3. **It seems the histopathological characteristics are available for all cases included in this study. Such information should be included in the descriptive analysis**

This descriptive data is now included in the demographics table (Table 1) as illustrated below.

| **Tumour Histology** | Combined n(prop) | HIV – n(prop) | HIV + n(prop) | P |
| --- | --- | --- | --- | --- |
| Squamous Cell | 207 (0.80) | 115 (0.83) | 92 (0.77) | **Ref.** |
| Adenocarcinoma | 25 (0.10) | 14 (0.10) | 11 (0.09) | 0.950^b^ |
| Adenosquamous | 11 (0.04) | 2 (0.01) | 9 (0.08) | **0.017** ^b^ |
| Other* | 15 (0.06) | 8 (0.06) | 7 (0.06) | 0.900^b^ |

Other^*^**=** spindle cell carcinoma, papillary serous carcinoma, adenoid cystic, adenosarcoma, small cell carcinoma.

4. **Maybe only relevant covariables to the current study are needed to be described. Some of the information collected through the project but not used for the study might not be necessary to mention, such as weight, height, comorbidities and treatment etc.**

Thank you for this observation. All variables that are not further analysed in this study have been deleted. The amended statement reads:

Demographic information such as age, residency, histopathological tumour characteristics, were collected from the patient folder in the RTC. In addition, behavioral and lifestyle factors were collected by interviewing the study participants, namely history of alcohol, smoking and parity. Sexual history information including age at sexual debut, number of sexual partners, history of circumcised partner, sexually transmitted infections were also collected. Data on HIV status was also collected for comparative analysis.

5. **I figure no significant association was detected in the univariate regression between HPV-related risk factors (i.e., age, sexual debut, parity and STI history) and any HR-HPV genotypes might be due to limited power, when the HR-HPV types were stratified by each type. However, those are known factors that associated with HPV infection. Could the authors provide estimates (figure 3b) additionally adjusting for those factors and histology at least as sensitivity analysis when examine the co-occurrence of HPV types. Besides, please clarify what is the reference group for the ORs.**

As observed by the reviewer, these analyses had not been performed due to a lack of association detected in the univariate analysis. To ensure that these findings were not influenced by limited power of study, multivariable regression was performed using the HPV co-occurrences as predictors, and adjusting for the known HPV risk factors- age, sexual debut, parity, STI history and HIV status, and the findings were tabulated into Supplementary Table 3. Although there were no definitive indicators determined in this sensitivity analyses, we did observe a tendency towards of the HPV16/18 co-occurrence to be influences by age HIV status and age of sexual debut. A larger cohort would be needed to confirm the validity of the potential trend observed here.

**Supplementary Table 3. Multivariate regression analysis of co-occurring HPV genotypes with risk factors.**

| Co-variates | OR (95% CI) | P |
| --- | --- | --- |
| HPV16/18 |  |  |
| Age | 1.0 (1.0 – 1.1) | 0.684 |
| HIV | 2.3 (0.9 -5.6) | 0.065 |
| STI history | 0.5 (0.2 – 1.3) | 0.159 |
| Parity | 1.1 (0.9 – 1.4) | 0.274 |
| Age of sexual debut | 1.1 (1.0 – 1.3) | 0.076 |
| HPV16/33 |  |  |
| Age | 1.0 (0.9 – 1.0) | 0.547 |
| HIV | 0.4 (0.1 – 1.5) | 0.174 |
| STI history | 1.0 (0.2 – 3.9) | 0.956 |
| Parity | 1.0 (0.7 – 1.3) | 0.821 |
| Age of sexual debut | 1.0 (0.8 – 1.2) | 0.834 |
| HPV16/35 |  |  |
| Age | 1.0 (1.0 – 1.1) | 0.152 |
| HIV | 0.8 (0.4 – 1.6) | 0.573 |
| STI history | 1.1 (0.7 – 2.2) | 0.769 |
| Parity | 1.0 (0.9 – 1.2) | 0.985 |
| Age of sexual debut | 1.0 (0.9 – 1.2) | 0.532 |
| HPV35/51 |  |  |
| Age | 1.0 (1.0 – 1.1) | 0.282 |
| HIV | 1.0 (0.2 – 4.4) | 0.999 |
| STI history | 3.0 (0.8 – 12.2) | 0.115 |
| Parity | 0.9 (0.7 – 1.3) | 0.670 |
| Age of sexual debut | 1.0 (0.8 – 1.3) | 0.856 |
| HPV35/52 |  |  |
| Age | 1.0 (0.8 – 1.3) | 0.930 |
| HIV | NA | NA |
| STI history | NA | NA |
| Parity | 0.9 (0.3 – 3.4) | 0.933 |
| Age of sexual debut | 1.5 (0.7 – 3.6) | 0.332 |
| HPV39/51 |  |  |
| Age | 1.0 (0.8 – 1.2) | 0.827 |
| HIV | NA | NA |
| STI history | NA | NA |
| Parity | 1.2 (0.7 – 2.0) | 0.602 |
| Age of sexual debut | 1.0 (0.5 – 1.9) | 0.984 |
| HPV39/56 |  |  |
| Age | 1.2 (1.0 – 1.4) | 0.108 |
| HIV | NA | NA |
| STI history | NA | NA |
| Parity | 1.2 (0.7 – 2.3) | 0.520 |
| Age of sexual debut | 1.2 (0.7 – 2.3) | 0.582 |

6. **A legend explaining how the HR-HPV types were classified (ie. hierarchical classification) especially for those cases with multiple types of HPV infection in table 1 could be informative.**

Thank you for this recommendation. In our analysis, HPVs were only analysed at a genotype level, due to the limited coverage of the HPV genotyping assay used to test for HPV DNA. We have clarified this in the methods section of the manuscript, and have also included a figure legend in the results for reference to phylogenetic clades in the discussion section.

**Methods section:**

For analysis all individual HPV genotypes regardless of whether they occur as single or multiple infections were analysed were reported as standalone genotypes. None of the statistical analysis taking into account the species from which the HPV genotypes are from.

**Results section:**

**a= Human papillomavirus alpha 9 species; b= Human papillomavirus alpha 7 species; c= Human papillomavirus alpha 5 species; d= Human papillomavirus alpha 6 species.**

7. **In table 4, could the authors clarify the data source for the HPV types in each region of Africa if they are from external studies?**

Thank you for this comment. We have clarified in the results section where we obtained the data from. The prevalence of the various HPV genotypes in the Zimbabwean study cohort was compared to the most common HPV genotypes on other African women across the continent, using data obtained from HPV Information Centre database (<https://hpvcentre.net>).

8. **Could the authors provide a detailed descriptive table as supplement the distribution of HR-HPV types by HIV status for all included women (including the specific types for multiple infections)?**

The additional data for the HPV multiple infections by HIV status was collated into a supplementary table 2. Further inclusion of more than 2 HR-HPVs
