# Supplementary material for: High-risk HPV genotypes in Zimbabwean women with cervical cancer: Comparative analyses between HIV-negative and HIV-positive women
Source: PLoS One. 2021 Sep 28;16(9):e0257324. doi: 10.1371/journal.pone.0257324 (PMC8478215; doi:10.1371/journal.pone.0257324)
Supplement: Attachment — Submitted filename: PONE-D-21-04071R1-responses to reviewers 24082021.docx [file pone.0257324.s005.docx]

**Response to Reviewer Comments: PONE-D-21-04071R1**

24 August 2021,

Dear Professor Graciela Andrei

Thank you for reviewing our manuscript entitled “High-Risk HPV genotypes in Zimbabwean women with cervical cancer: Comparative Analyses between HIV-negative and HIV-positive women” and providing us with constructive criticism on how to improve the statistical analysis and the scientific soundness of the work. Below are our responses to each of the reviewers’ and editor’s comments;

**Reviewer #2: Thank you for the extensive work from the authors in addressing the comments, and manuscript has been substantially improved. However, there are a couple responses to the comments could be further clarified. In the response to comment point 5, the author has already performed a multivariate regression analysis, using the HPV genotypes as predictors, while controlling for age and HIV status, and the ORs for significant HPV genotypes were illustrated in fig 3b. Therefore, the sensitivity analysis is expecting to be repeating exact the same regression model by additionally control for factors of sexual debut, parity, STI history and histology and presented the ORs as a supplementary figure or table instead of presenting ORs for each of the covariate as shown in the current supplementary table 3.**

Thank you for further clarifying the expectations of the reviewer, which the authors may have misunderstood in the previous peer review process. The authors have done the best they can to address this comment. To this effect, S3 Table was added, consisting of multivariate analysis for the co-segregation of the HPV genotypes, taking into consideration the proposed covariates, namely, age, history of STI, parity, HIV status, age of sexual debut, tumour histology, as suggested. The comment was addressed in the manuscript to read:

In order to ensure that lack of significance observed was not a result of low study power, a sensitivity appraisal was conducted, using multivariate regression analyses controlling for age, history of sexually transmitted infections, parity, HIV status, age at sexual debut and tumour histology. In this sensitivity analyses, all the co-segregation patterns remained statistically significant except HPV16/33 (OR=0.4; 95% CI=0.2-1.9; p=0.05), HPV35/52 (OR=0.5; 95% CI=0.1-4.3; p=0.50) (S3 Table).

**Table S3. Sensitivity analysis for HPV co-segregation.**

| **HPV genotypes*** | **OR (95% CI)** | **P** |
| --- | --- | --- |
| 16/18 | 0.3 (0.1-0.5) | **<0.01** |
| 16/33 | 0.4 (0.2-1.0) | 0.05 |
| 16/35 | 3.6 (1.8-7.2) | **<0.01** |
| 35/51 | 8.8 (2.3-34.3) | **<0.01** |
| 35/52 | 0.5 (0.1-4.3) | 0.50 |
| 39/51 | 4.2 (0.7-25.9) | 0.12 |
| 39/56 | 9.5 (1.1-82.9) | **0.04** |

*multivariate analysis for the co-segregation of HPV genotypes, taking into consideration age, history of STI, parity, HIV status, age of sexual debut, tumour histology.

**I appreciate the work done by the authors for supplementary table 2, however, what would be informative to address the comments point 8 is to provide a simple frequency table showing the distribution of hrHPV types, including both single type infection and multiple types of infection by HIV status (HIV positive and HIV negative). Considering there are a proportion of women had more than 2 types of HPV infection (table 2), showing the actual HPV types by HIV status could be interesting to the readership.**

Thank you for indicating which analyses and data would be more interesting to the readers. The comments were received well and taken into consideration. The authors have amended the table to report on absolute frequencies, by HIV status, as shown below. This tabulation was favoured as it clearly represents the number of individuals with specific HPV genotypes depending on HIV status.

**S2 Table. The number of individuals harbouring specific HPV genotypes stratified by HIV status.**

|  |  | **Number of HPVs (n)** | | | | | |
| --- | --- | --- | --- | --- | --- | --- | --- |
| **HPV genotype** | **HIV status** | **1** | **2** | **3** | **4** | **5** | **6** |
| 16 | - | 19 | 18 | 5 | 1 | 0 | 0 |
|  | + | 15 | 13 | 3 | 1 | 1 | 0 |
| 18 | - | 13 | 12 | 1 | 0 | 1 | 1 |
|  | + | 6 | 13 | 1 | 0 | 0 | 0 |
| 31 | - | 1 | 0 | 1 | 2 | 0 | 0 |
|  | + | 1 | 0 | 0 | 0 | 0 | 1 |
| 33 | - | 2 | 0 | 1 | 2 | 0 | 0 |
|  | + | 2 | 0 | 0 | 0 | 0 | 0 |
| 35 | - | 15 | 15 | 3 | 0 | 1 | 0 |
|  | + | 3 | 10 | 3 | 0 | 1 | 1 |
| 39 | - | 0 | 2 | 0 | 1 | 0 | 0 |
|  | + | 0 | 1 | 0 | 0 | 0 | 0 |
| 45 | - | 1 | 0 | 2 | 3 | 0 | 0 |
|  | + | 0 | 0 | 1 | 1 | 0 | 0 |
| 51 | - | 0 | 0 | 1 | 0 | 1 | 0 |
|  | + | 1 | 1 | 1 | 1 | 1 | 1 |
| 52 | - | 1 | 2 | 1 | 0 | 0 | 0 |
|  | + | 0 | 3 | 1 | 0 | 0 | 1 |
| 56 | - | 0 | 0 | 0 | 1 | 0 | 0 |
|  | + | 0 | 0 | 1 | 0 | 1 | 0 |
| 58 | - | 0 | 2 | 1 | 1 | 1 | 0 |
|  | + | 0 | 2 | 0 | 0 | 1 | 0 |
| 59 | - | 0 | 0 | 0 | 2 | 0 | 0 |
|  | + | 2 | 0 | 1 | 1 | 0 | 0 |
| 66 | - | 0 | 0 | 0 | 1 | 0 | 0 |
|  | + | 0 | 0 | 1 | 0 | 0 | 0 |
| 68 | - | 2 | 0 | 1 | 0 | 1 | 0 |
|  | + | 1 | 0 | 1 | 1 | 2 | 1 |
| **TOTAL** |  | 85 | 94 | 31 | 19 | 12 | 6 |
